# Proximal Humerus Fractures: A Review of Anatomy, Classification, Management Strategies, and Complications

**DOI:** 10.7759/cureus.73075

**Published:** 2024-11-05

**Authors:** Zubair Younis, Muhammad A Hamid, Jebran Amin, Muhammad Murtaza Khan, Gurukiran Gurukiran, Rahul Sapra, Rohit Singh, Kubra Farooq Wani, Zuhaib Younus

**Affiliations:** 1 Orthopaedics, The Royal Wolverhampton NHS Trust, Wolverhampton, GBR; 2 Orthopaedic Surgery, University Hospitals Birmingham, Birmingham, GBR; 3 Trauma and Orthopaedics, Ysbyty Gwynedd Hospital, Bangor, GBR; 4 Trauma and Orthopaedics, Princess Royal Hospital Telford, Telford, GBR; 5 Orthopaedics and Trauma, Royal Shrewsbury Hospital, Shrewsbury, GBR; 6 Trauma and Orthopaedics, Walsall Manor Hospital, Walsall, GBR; 7 Orthopaedics, Shrewsbury and Telford Hospitals NHS Trust, Shrewsbury, GBR; 8 Psychiatry, The Redwoods Centre, Shrewsbury, GBR; 9 Pulmonology and Critical Care, Government Medical College, Srinagar, Srinagar, IND

**Keywords:** classification systems, conservative management, proximal humerus fractures, reverse shoulder arthroplasty (rsa), surgical treatment

## Abstract

Proximal humerus fractures are prevalent in older adults, particularly women, primarily due to osteoporosis and increased fall risk. These fractures often result from low-energy falls in elderly patients, while in younger individuals, they are more likely to occur with high-energy trauma, which may involve additional injuries to soft tissue and neurovascular structures. Proper anatomical understanding, including key structures and blood supply, is crucial for effective management and to prevent complications. Several classification systems assist in guiding treatment for proximal humerus fractures, including Codman’s, Neer's, Arbeitsgemeinschaft für Osteosynthesefragen/Orthopaedic Trauma Association (AO/OTA) system, and the Codman-Hertel system, which helps predict ischemia risk.

Evaluation of proximal humerus fractures begins with Advanced Trauma Life Support (ATLS) protocols, emphasizing a thorough shoulder assessment, particularly focusing on skin integrity in elderly patients. Neurological and vascular examinations are essential due to the common occurrence of nerve injuries, especially involving the axillary nerve. Imaging typically includes multiple standard views, with advanced imaging reserved for complex cases and for assessing associated soft tissue injuries. Treatment options range from conservative management for stable fractures to surgical intervention for more complex cases. Surgical choices include techniques like fixation, nailing, and various arthroplasty options, with some procedures potentially offering advantages for older adults with bone quality or soft tissue challenges. Rehabilitation is a vital component of recovery, with emphasis on early mobility and gradual strengthening to restore function, especially in older patients.

Complications following open reduction and internal fixation (ORIF) for proximal humerus fractures can include issues such as non-union, malunion, osteonecrosis, infection, joint stiffness, and fixation failure. In cases where non-union or fixation failure occurs, revision surgery or arthroplasty may be necessary. Joint stiffness may require further intervention if physical therapy is insufficient, while symptomatic osteonecrosis might also need surgical management. Malunion is generally better tolerated in older patients but may require correction in younger individuals. Other surgical options, such as hemiarthroplasty (HA) and reverse shoulder arthroplasty (RSA), share similar risks, including infection, fractures, complications at the tuberosity, stiffness, and instability. RSA may be favored when there are tuberosity or rotator cuff issues. Closed reduction with percutaneous pinning carries a high risk of pin migration and malunion, which can result in deformities, pain, and dysfunction. Proper anatomical knowledge is essential to avoid neurovascular injury and to manage common issues such as pin-site infections effectively.

## Introduction and background

Proximal humerus fractures account for approximately 5-6% of all fractures, representing the most common humeral fracture [1,2]. The incidence of proximal humerus fractures varies by region, with an analysis in southern Europe showing an incidence rate of 60.1 fractures per 100,000 person-years from 2016 to 2018 [3]. Similarly, in Australia, the incidence was reported as 45.7 fractures per 100,000 person-years in 2017 [4]. In southern Europe, the rate among women was notably higher at 89.3 per 100,000 person-years, compared to 28.2 in men [3]. In Australia, women over 85 had the highest incidence, reaching 711.8 fractures per 100,000 person-years in 2017 [4].

Over 70% of proximal humerus fracture cases involve patients aged 60 or older, with women comprising 75% of this group [1,5]. This higher incidence among elderly is often attributed to osteoporosis and an increased risk of falls, typically low-energy falls from standing height [5,6]. Conversely, proximal humerus fractures in younger populations are usually related to high-energy trauma, often accompanied by concomitant soft tissue and neurovascular injuries [7].

Studies within preselected cohorts suggest an incidence range of 60.1 to 90.8 cases per 100,000 person-years, though variability stems from differences in reporting methods and regional population health [8]. There remains an ongoing need for comprehensive data, as certain populations may be underrepresented in current literature.

When it comes to treatment, the ideal approach for proximal humerus fractures remains a topic of debate, with recent advancements prompting a shift from conservative management to more individualized, patient-centered care. A Cochrane review of 23 randomized trials involving 1,238 patients concluded that there is currently insufficient evidence to definitively guide treatment decisions for these fractures [9]. Factors such as injury classification, patient age, and medical history heavily influence the surgeon’s choice of treatment [10]. Given the range of options available, informed, patient-specific discussions that consider the patient’s unique circumstances and the surgeon's technical expertise are essential to optimizing outcomes.

This review provides an in-depth look at the anatomy, classification, treatment approaches, and outcomes for proximal humerus fractures. The following sections will cover these aspects, exploring both traditional and emerging insights in the management of these injuries.

## Review

Anatomy

**Figure 1:**
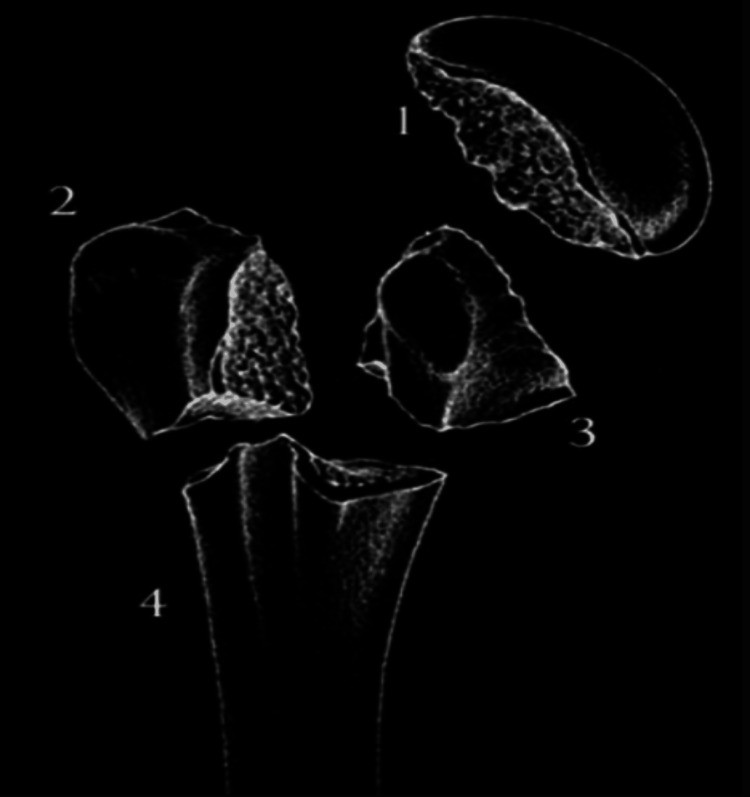
Segments of the proximal humerus as per Neer’s classification 1: Humeral Head 2: Greater Tuberosity 3: Lesser Tuberosity 4: Humeral shaft Source: Orthobullets, reproduced with permission

**Figure 2:**
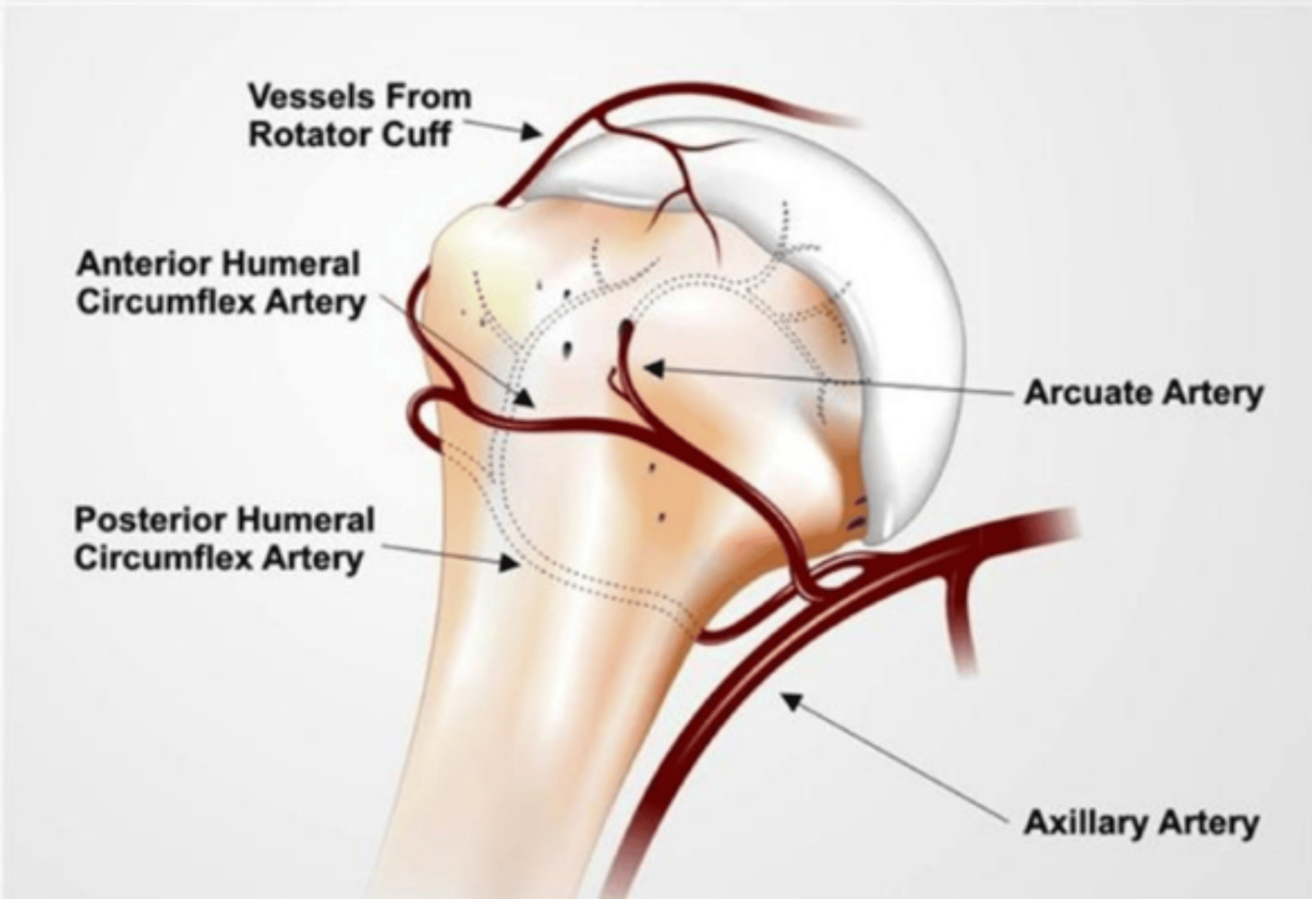
Vascularity of the humeral head Source: Orthobullets, reproduced with permission

The proximal humerus can be divided into various anatomic landmarks such as the anatomical and surgical neck, as well as the greater and lesser tuberosities (Figure 1). The average neck shaft angle of the proximal humerus is 135 degrees. Recent studies have indicated that the major blood supply for proximal humerus comes from the posterior circumflex humeral artery [11]. The remaining blood supply comes from the anterior circumflex humeral artery, which is a branch of axillary artery (Figure 2). The arcuate artery, a branch of anterior circumflex humeral artery, contributes a major portion of the blood supply to the greater tuberosity. There is also some contribution from blood vessels of the rotator cuff.

In proximal humerus fractures, certain anatomical features serve as key predictors of ischemia of the humeral head. Good predictors of ischemia include the length of the metaphyseal head extension, the integrity of the medial hinge, and the basic fracture pattern [12]. Moderate and poor predictors of ischemia include fractures consisting of four fragments, angular displacement of the head, the amount of displacement of the tuberosities, glenohumeral dislocation, head-split components, and fractures consisting of three fragments. When high-risk features such as a short calcar, disrupted medial hinge, and involvement of the anatomical neck are combined, predictive values for ischemia can reach as high as 97% [12].

Classification

It was Ernest Codman in 1934 who classified proximal humeral fractures into four anatomic parts: humeral shaft, articular surface, greater tuberosity, and lesser tuberosity [13].

In 1970, Charles Neer introduced a classification system for proximal humeral fractures, which remains one of the most commonly used classifications today [14]. Codman's earlier classification was limited by its lack of detail regarding fracture displacement and angulation, a shortcoming that Neer addressed [15]. According to Neer's classification, displacement is defined as a fracture fragment that is separated by more than 1 cm or angulated by more than 45 degrees [5]. Neer expanded upon Codman’s use of the four main anatomic segments, while also quantifying and qualifying fracture displacement. Using this definition of displacement, Neer defined fractures as one-part, two-part, three-part, or four-part [5].

**Figure 3:**
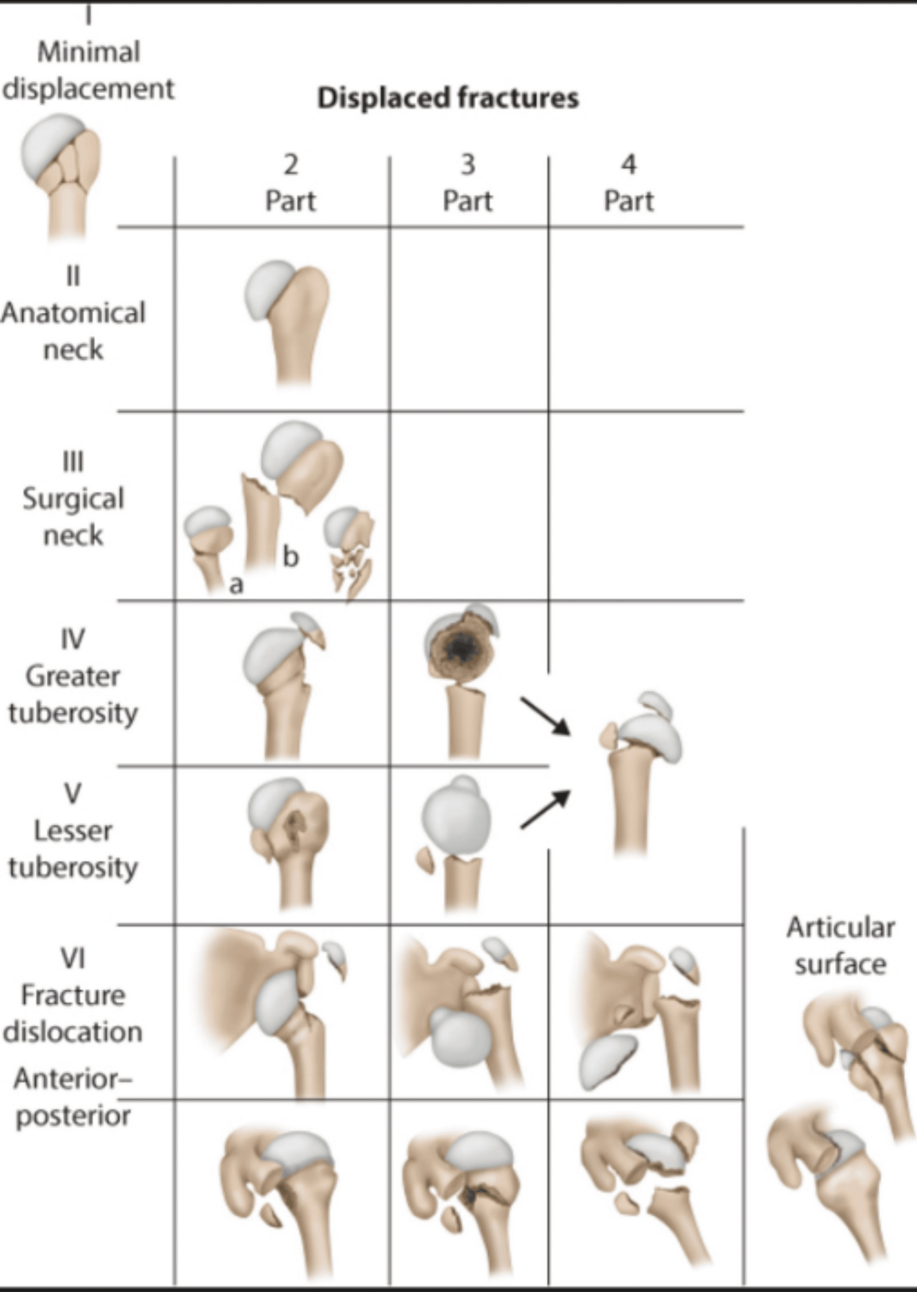
Neer’s classification of proximal humerus fractures Source: Orthobullets, reproduced with permission

The proximal humerus, according to Neer, is divided into four parts: the humeral head (articular segment), the greater tuberosity, the lesser tuberosity, and the shaft of the humerus. As per Neer, all undisplaced fractures are categorised together, irrespective of the number of fragments while displaced fractures are divided into specific groups. Neer applied this method to classify fractures into six categories: I - minimally displaced fractures, II - displaced anatomic neck fractures, III - displaced surgical neck fractures, IV - displaced greater tuberosity fractures, V - displaced lesser tuberosity fractures, and VI - fracture-dislocations (Figure 3) [5].

**Figure 4:**
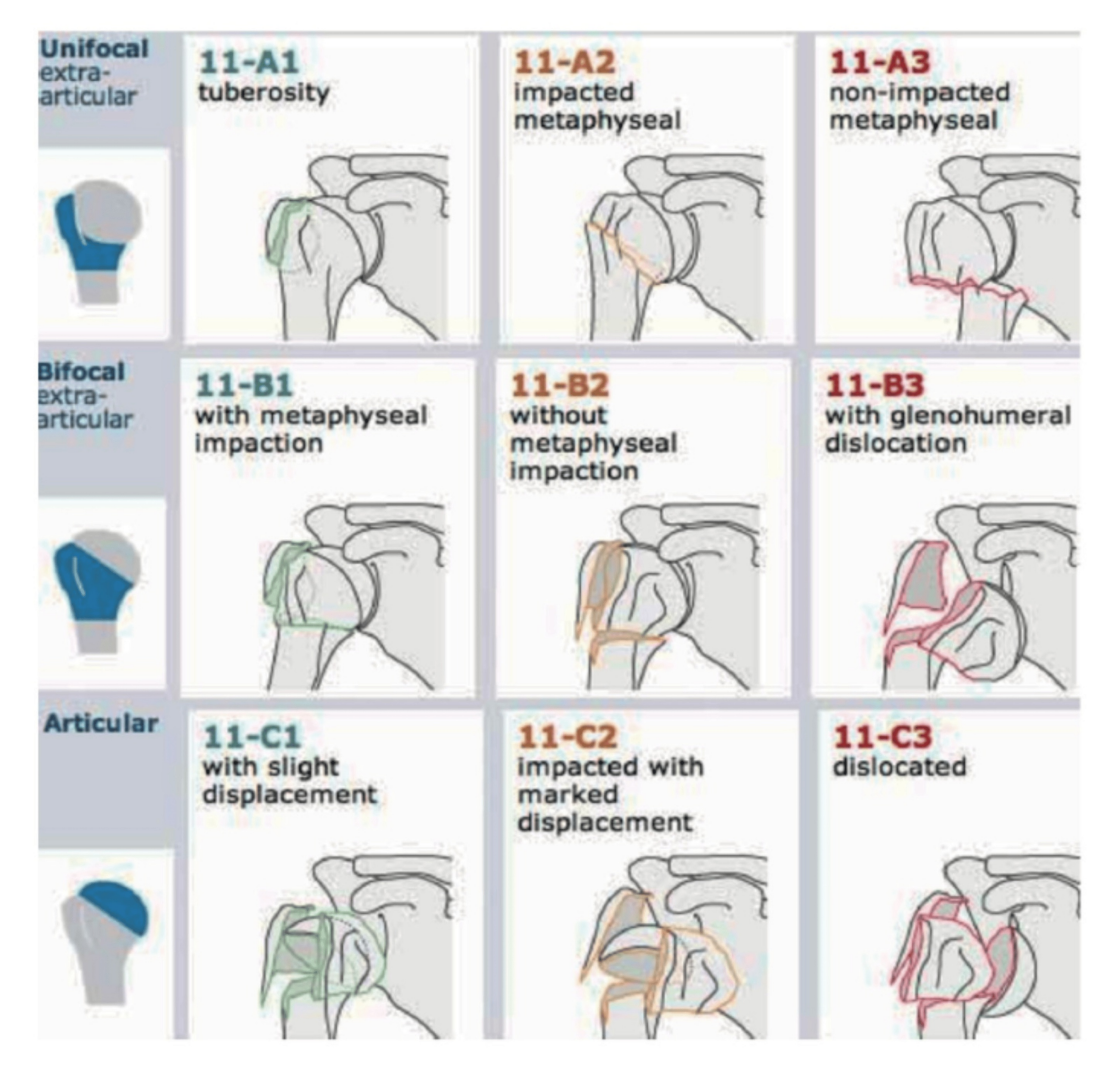
Arbeitsgemeinschaft für Osteosynthesefragen/Orthopaedic Trauma Association (AO/OTA) proximal humerus fractures classification Reprinted with permission. Copyright by AO foundation, Switzerland. Source: AO surgery reference, www.aosurgery.org

The Arbeitsgemeinschaft für Osteosynthesefragen/Orthopaedic Trauma Association (AO/OTA) classification is a widely used system for classifying proximal humerus fractures, based on the fracture's location, type, and severity [16]. This system is more detailed than Neer's classification and is based on both the number of fracture fragments and the complexity of the fracture. Moreover, it divides proximal humerus fractures into three main types: Type A (extra-articular, unifocal fractures), which includes simple fractures of the greater tuberosity, lesser tuberosity or surgical neck; Type B (extra-articular, bifocal fractures), involving multiple locations like the tuberosity and surgical neck or with impaction; and Type C (articular fractures), which are more complex and involve the articular surface of the humeral head with varying degrees of comminution (Figure 4). Each type is further subdivided based on fracture complexity, which helps to guide treatment and prognosis. Importantly, the OTA classification identifies the importance of valgus impaction of the proximal humeral neck which is a distinct fracture pattern not included in previous classification systems [5].

**Figure 5:**
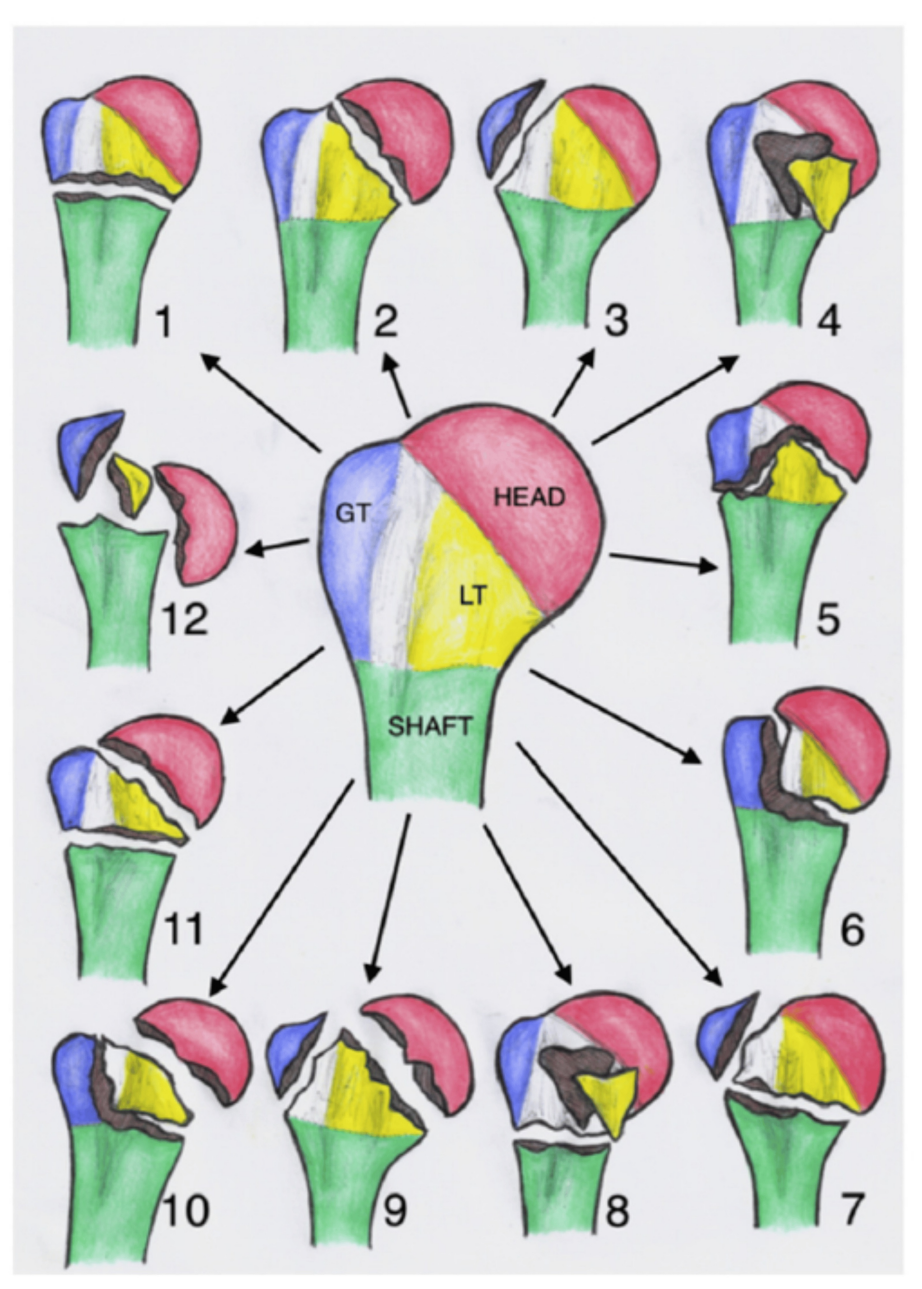
Modified description of Hertel’s binary or Lego description system for proximal humeral fractures, showing the 12 possible fracture patterns. GT, greater tuberosity; LT, lesser tuberosity Reproduced with permission [18].

In 2004, Hertel et al. developed the Codman-Hertel classification, a binary "lego-block" system designed to identify factors predicting humeral head ischemia caused by proximal humerus fractures (Figure 5). Hertel’s team emphasized the significance of fracture morphology, particularly the disruption of the medial hinge and involvement of the medial calcar (<8 mm), as key indicators of fracture fragment viability and head vascularity [12]. They concluded that the most relevant predictors of ischemia were the length of the dorsomedial metaphyseal extension, the integrity of the medial hinge, and the basic fracture type determined through this binary description system. Building on Codman’s earlier work, the system used structured questionnaires to classify proximal humerus fractures into 12 basic types. Similarly, Resch et al. introduced a related classification, focusing on valgus versus varus impacted fractures [17].

Table 1 provides a side-by-side comparison of key classification systems for proximal humerus fractures, outlining their basis, features, and implications for treatment. This comparative overview aids clinicians in selecting the most relevant system based on fracture characteristics and clinical applicability.

**Table 1:** Comparison of Classification Systems for Proximal Humerus Fractures AO/OTA: Arbeitsgemeinschaft für Osteosynthesefragen/Orthopaedic Trauma Association

Classification System	Basis of Classification	Key Features	Implications for Treatment
Codman	Anatomical landmarks	4-part model	Limited guidance; used mainly for localization
Neer	Displacement and number of fragments	1-4 parts based on displacement (≥1 cm or >45°)	Undisplaced fractures irrespective of parts often treated conservatively; Displaced fractures may require surgery
AO/OTA	Location and articular involvement	Types A, B, C, with subdivisions	Detailed fracture patterns aid in surgical planning, especially for complex fractures
Codman-Hertel	Fracture morphology & ischaemia prediction	Emphasizes medial hinge integrity and calcar length	Useful for predicting ischemia and assessing vascular risk for surgical planning

Clinical presentation and radiology

Patients are clinically evaluated based on the nature of the injury, with the initial assessment typically following the Advanced Trauma Life Support (ATLS) protocol unless the injury is isolated to the shoulder. The evaluation should start with a thorough history and examination including inspection of the soft tissues and skin, particularly in elderly patients who have more delicate tissues and are prone to impaired wound healing [19]. The primary focus during evaluation is identifying any neurological or vascular deficits or any open injuries associated with the fracture. Neurologic deficits, such as axillary nerve dysfunction or brachial plexus injuries, are common with these fractures, though assessment is difficult as the patient’s limited mobility hinders movement testing. In such cases, testing of sensation over the regimental badge area for axillary nerve may be the only available method, but this too can be affected by swelling and bruising. Regarding nerve injury, Visser et al. used electromyography to quantify the incidence of nerve lesion in proximal humerus fractures [20]. They found that 67% of patients exhibited nerve denervation following a proximal humerus fracture. The axillary, suprascapular, and radial nerves were the most affected.

**Figure 6:**
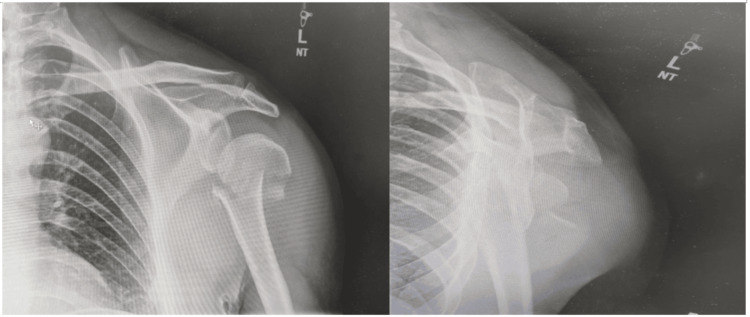
Anteroposterior and Scapular Y views demonstrating a fracture of proximal humerus Image Credits: First Author

**Figure 7:**
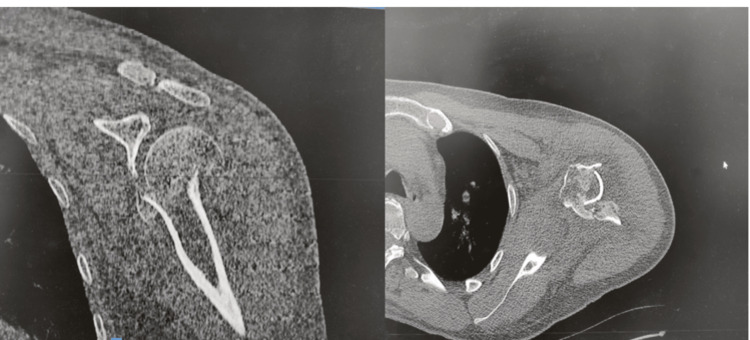
CT images demonstrating a fracture of proximal humerus Image Credits: First Author

Radiological imaging plays a crucial role in determining the fracture configuration. Standard radiographs for assessing a possible proximal humerus fracture include anteroposterior views (Grashey views), axillary lateral views, and scapular Y views (Figure 6). However, pain from the fracture can make it difficult to obtain axillary lateral views, and as such Velpeau views may be considered as an alternative. CT scans can help to delineate the fracture pattern and facilitate surgical planning, particularly in complex fracture dislocations with significant comminution (Figure 7) [19]. While MRI is less commonly used for routine fractures, it may be employed when soft tissue injuries or associated injuries, such as rotator cuff tears, are suspected. MRI provides detailed images of soft tissues and aids in assessing the extent of soft tissue damage [21].

Management

Treatment for proximal humerus fractures has always been controversial, with no general consensus reached on the methods of treatment in different age groups and different fracture configurations. A Cochrane review of 23 randomized trials involving 1,238 patients concluded that there is insufficient evidence to guide the treatment of these fractures [9]. The available treatment options for these fractures include conservative management and surgical intervention.

Conservative Management

This approach is recommended for non-displaced fractures, minimally displaced fractures involving the tuberosities, fractures in elderly or frail patients, as well as in low-demand individuals and those considered poor surgical candidates. Patients are typically placed either in a collar and cuff or an arm sling for three weeks, followed by mobilization in the form of progressive physical therapy rehabilitation to prevent stiffness [22].

The PROFHER trial, the largest randomized pragmatic study on proximal humerus fractures, concluded that for patients with displaced proximal humeral fractures involving the surgical neck, there was no significant difference in patient-reported clinical outcomes between operative and nonoperative treatments two years after the date of injury when the fracture was sustained [23].

Overall proximal humerus fractures in the elderly patients, are often considered fragility fractures. Treating surgeons should recommend vitamin D supplementation for all patients presenting with an acute proximal humerus fracture and consider referral to appropriate services for review of their bone health [5].

Operative Management

This mode of treatment is preferred in fractures that are displaced, comminuted, or angulated in patients who are suitable candidates for surgery. The various methods of operative treatment include open reduction internal fixation (ORIF), intramedullary nailing, hemiarthroplasty (HA), reverse shoulder arthroplasty (RSA), and closed reduction and percutaneous pinning.

ORIF: Open reduction and internal fixation is an effective method of treatment for proximal humerus fractures [24]. It is commonly used for displaced three-part and four-part fractures and valgus-impacted four-part humerus fractures to promote early shoulder movement [25-27]. Various fixation methods have been proposed, including tension band fixation [15,28], conventional large fragment and small fragment plates and screws [26,29,30] and locked plate and screw constructs [31-34].

**Figure 8:**
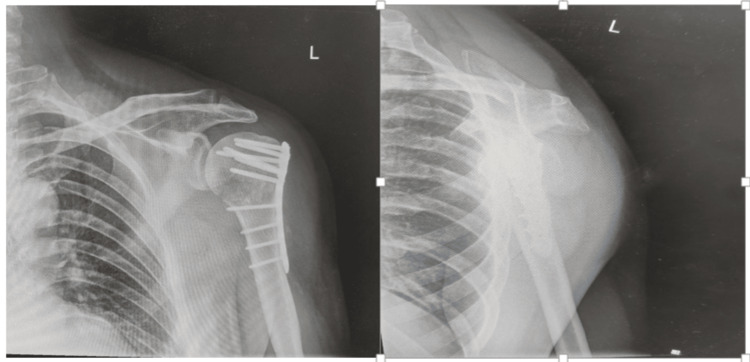
Anteroposterior and scapular Y view radiographs post open reduction internal fixation (ORIF) following a proximal humerus fracture Image Credits: First Author

The surgical approach will depend on the fracture pattern and surgeon preference, with options including a deltopectoral or deltoid-splitting approach. Articular fractures should be anatomically reduced, and relationships of the tuberosities and their associated rotator cuff insertions should be restored (Figure 8) [24]. When plating complex fractures, suture fixation of the tuberosities and medial augmentation with cement, bone graft, and calcar screws is suggested [19].

In patients with osteoporotic bone, a 6-8cm segment of fibula, a fibular graft strut can be placed in an intramedullary fashion to augment locking plate constructs. The graft is positioned proximal to the surgical neck and secured with screws to reduce the medial cortex and provide calcar support [32].

Gomberwalla et al. performed a meta-analysis of data from 12 studies comparing ORIF and arthroplasty for treating three- and four-part fracture patterns. The analysis showed that ORIF led to significantly higher Constant scores than arthroplasty, suggesting better functional outcomes [35]. However, significant heterogeneity among the studies included limits the conclusions that can be drawn.

Intramedullary nailing: The use of intramedullary nails are effective in stabilising some proximal humerus fractures, which include surgical neck fractures, three-part greater tuberosity fractures in the young, and combined proximal humerus and humeral shaft fractures [36]. Intramedullary nails have a theoretical advantage of a smaller incision with relatively less blood loss. There have been some concerns with the use of intramedullary nails, particularly with potential damage to the rotator cuff muscles and proximal screw migration. This has been addressed in newer implants with a change in geometry and proximal locking constructs [5,37].

Hemiarthroplasty: Historically, shoulder HA has been the gold standard for the treatment of comminuted four-part proximal humerus fractures not suitable to ORIF in the elderly population [38]. In recent years, RSA has become increasingly popular for these patients [39,40]. Comparing HA and RSA implantation for proximal humeral fractures shows that RSA generally yields more favourable outcomes, whereas HA outcomes are less predictable [41-43]. However, hemiarthroplasty still has its place, particularly for younger patients with unreconstructable fractures, those with axillary nerve injuries affecting deltoid function, and individuals with compromised glenoid bone stock that cannot be managed with glenoid resurfacing or glenosphere placement [44,45].

**Figure 9:**
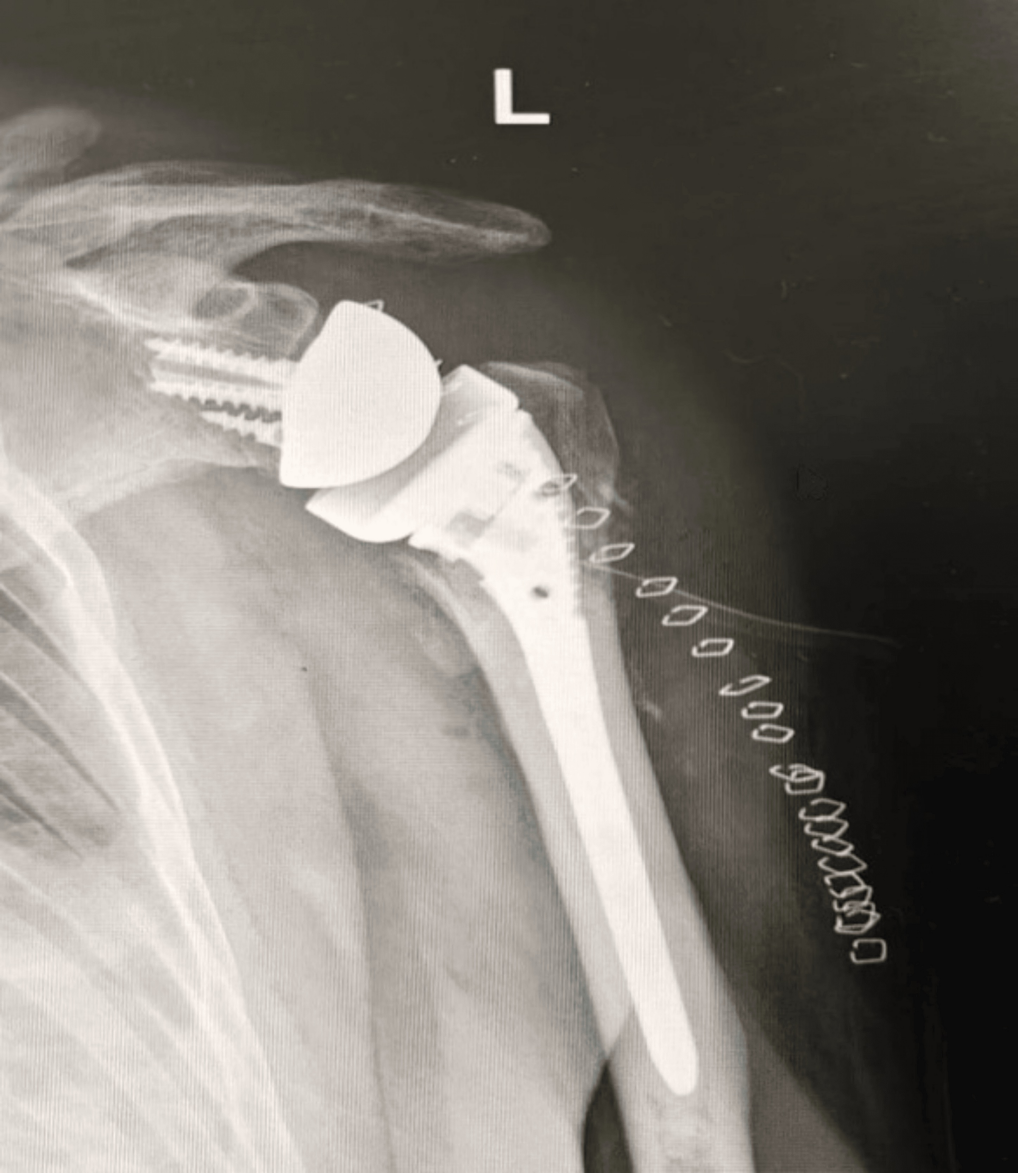
Post operative anteroposterior radiograph after reverse shoulder arthroplasty (RSA) Image Credits: First Author

Reverse shoulder arthroplasty: RSA is indicated in displaced three- or four-part proximal humerus fractures in elderly patients with poor bone stock, it is also appropriate in cases of proximal humerus fractures with irreparable rotator cuff damage. Additionally, it is considered when conservative treatment has failed, leading to complications such as non-union or malunion [46,47]. RSA is especially advantageous in these cases, as it compensates for rotator cuff dysfunction and improves pain relief while restoring shoulder function. RSA is relatively indicated for fractures when there is a high risk of poor outcomes from plate osteosynthesis or hemiarthroplasty due to factors such as irreparable fractures, potential humeral head osteonecrosis, compromised tuberosity bone quality, and preexisting chronic rotator cuff tears or arthritis (Figure 9) [48]. Absolute contraindications for RSA include permanent axillary nerve dysfunction, global deltoid muscle dysfunction, and global brachial plexopathy. However, if the brachial plexus injury is limited to the lower nerve roots with sparing of axillary nerve function, RSA may still be feasible [48]. Partial deltoid dysfunction is a relative contraindication, but RSA may still provide reasonable results [49]. Relative contraindications also include acromion or scapular spine fractures that could displace under deltoid tensioning, as well as glenoid fractures or deficiencies that prevent stable baseplate fixation. Arthroplasty should be approached with extreme caution in cases of open fractures due to the heightened risk of infection. Additionally, the inability to adhere to postoperative restrictions and rehabilitation protocols, along with significant medical comorbidities, are also considered relative contraindications [48].

Recent meta-analyses indicate that RSA consistently outperforms both HA and ORIF for proximal humeral fractures. Chen et al. found that RSA provides better functional outcomes, fewer complications, and lower rates of additional surgeries compared to HA and ORIF [50]. Additional meta-analyses of randomized controlled trials support RSA’s superiority over HA, with better forward flexion, abduction, and higher Constant scores [51]. The DelPhi study also highlighted RSA’s advantage, showing a 13.4-point improvement in Constant scores over ORIF, though with only two years of follow-up [52]. While RSA shows higher overall complication rates, its reoperation rate remains similar to HA, and its revision rate is lower.

Closed reduction and percutaneous pinning: While historically recognized as a viable technique, this method is rarely used in current practice and is primarily noted here for historical context. It can be highly effective for unstable two-part surgical neck fractures and even certain three-part or four-part fractures in patients with strong bone quality [53]. Closed reduction is performed, followed by the placement of two pins from the greater tuberosity to the calcar and three or four pins from the lateral cortex into the humeral head. The pins are positioned divergently to maximise the strength of the construct. This treatment approach can be highly effective when selected for the appropriate patient group. Knowledge of the anatomy of the axillary and musculocutaneous nerves is essential in avoiding injury to these structures [54].

Rehabilitation

Rehabilitation plays a vital role in achieving the best possible outcomes for patients recovering from proximal humerus fractures. This comprehensive approach includes restoring range of motion, rebuilding strength, facilitating a gradual return to daily activities as well as pain management. Rehabilitation for proximal humerus fractures begins with early passive range of motion (ROM) exercises aimed at maintaining joint flexibility while protecting healing tissues [8]. In the immediate postoperative period, patients are encouraged to perform gentle shoulder shrugs, elbow, wrist and hand ROM exercises to prevent stiffness. After three weeks, shoulder ROM exercises are introduced, progressing to active-assisted movements under therapist supervision to gradually improve mobility [55]. Scapular mobility exercises are also emphasized to ensure coordinated shoulder movement. As recovery continues, patients are guided from isometric exercises, which activate shoulder muscles without undue joint strain, to resistance training to build shoulder strength [8]. Later stages of rehabilitation emphasize functional training, with exercises that mimic daily tasks like reaching, lifting, and overhead movements, helping patients regain practical mobility.

In elderly patients with complex proximal humerus fractures, rehabilitation poses unique challenges, demanding thoughtful, individualized strategies to address their specific needs. Rehabilitation plans are carefully tailored to each patient’s goals and lifestyle needs to ensure a safe, gradual return to daily activities. In elderly patients with complex proximal humerus fractures, rehabilitation poses unique challenges, demanding thoughtful, individualized strategies to address their specific needs. Rehabilitation plans are carefully tailored to each patient’s goals and lifestyle needs to ensure a safe, gradual return to daily activities.

Complications

ORIF

The most common complications following ORIF for proximal humerus fractures include non-union, malunion, osteonecrosis, infection, arthrofibrosis, and fixation failure.

Non-union occurs in 5-10% of cases and often arises from a mechanically unstable fracture pattern that has been poorly reduced and fixed or due to inadequate surgical technique. Patients present with persistent postoperative pain, decreased range of motion, and some functional loss. Plain radiographs typically reveal bony resorption and poor healing [5]. Treatment options include revision ORIF with bone grafting or conversion to shoulder arthroplasty, particularly if the non-union is accompanied by severe pain or functional impairment [56].

Arthrofibrosis is another possible complication, often presenting with stiffness and limited range of motion within months postoperatively. Initial treatment typically includes an intensive physical therapy regimen, which is successful for most patients. If range of motion fails to improve by six months, manipulation under anesthesia is recommended. If this fails, an arthroscopic rotator interval and capsular release can be performed to improve mobility [56].

Osteonecrosis has been reported in up to 15% of patients after ORIF for proximal humeral fractures, particularly in complex fracture patterns or in cases requiring extensive soft tissue stripping [56]. Patients with osteonecrosis often respond to physical therapy, but as the condition progresses, advanced collapse with severe symptoms may occur. In such cases, conversion to shoulder arthroplasty should be considered to alleviate pain and improve function.

Malunion is commonly well tolerated in elderly patients with limited functional demands but can be problematic in younger patients. In these patients, malunion may lead to shoulder dysfunction, subacromial impingement, and potential rotator cuff tears. Corrective osteotomy or conversion to arthroplasty may be considered for younger patients experiencing severe symptoms from malunion [5].

Infection rates are generally low but may occur superficially or deeply. Superficial infections typically resolve with oral antibiotics. In cases of deep infection with stable hardware, treatment involves incision, debridement, and antibiotic therapy while preserving the fixation. However, if the infection persists despite hardware retention and prolonged antibiotic therapy, a more aggressive debridement with implant removal is indicated, with delayed reconstruction planned for afterward [5].

Fixation failure is often due to poor bone quality or insufficient fixation. Revision ORIF may be attempted if bone stock allows, but in cases where fixation is not feasible, conversion to arthroplasty is generally recommended, particularly for patients experiencing significant functional limitations or pain.

Hemiarthroplasty and Reverse Shoulder Arthroplasty

Complications of shoulder HA and RSA include infection, intraoperative fractures, tuberosity nonunion or malunion, stiffness, heterotopic ossification, and instability [10]. In addition to these general complications, shoulder HA carries specific risks such as rotator cuff dysfunction and glenoid erosion or arthrosis. The incidence of infection in HA is reported to be 1.55% for superficial and 0.64% for deep infections [57]. Management of superficial and acute deep infections typically involves irrigation, debridement, and culture-guided antibiotics [58]. Chronic deep infections may require implant removal, the placement of an antibiotic spacer, prolonged IV antibiotic therapy, and delayed reimplantation [58].

Glenoid arthrosis is more commonly observed in younger and middle-aged patients who subject their shoulders to higher demands, often resulting in wear of the glenoid cartilage, lower functional outcomes, and in some cases, the need for conversion to total shoulder arthroplasty (TSA) [59]. Tuberosity complications are the most common complications following shoulder HA, with Boileau et al. reporting tuberosity malposition in 50% of HA cases [60]. Tuberosity malposition is frequently associated with rotator cuff dysfunction, both of which detrimentally impact functional outcomes. Proper prosthetic version and height selection are critical to prevent these complications [10]. When significant tuberosity or rotator cuff issues arise, revision to reverse total shoulder arthroplasty may be necessary.

Neurological complications are another consideration, with the axillary nerve being the most commonly affected, although other brachial plexus branches can also be injured. Boileau et al. found that 4% of HA patients experience nerve injury, although most cases are transient and resolve with observation and therapy [58].

Closed Reduction and Percutaneous Pinning

Pin migration is a frequent complication after closed reduction and percutaneous pinning of proximal humerus fractures and can lead to severe consequences [61]. Therefore, a good understanding of the anatomy is essential in performing this procedure. If these pins become loose, there is a risk of injury to important neurovascular structures. Additionally, post-operative pin-site infections are common, ranging from superficial to deep infections. Malunion is a more frequent complication with this fixation method compared to non-union [62]. Varus angulation of the articular surface and postero-superior displacement of the greater tuberosity are among the most common form of malunions. This can lead to subacromial impingement, pain, and loss of function [62]. If closed reduction and percutaneous pinning is attempted in elderly patients, there is a risk of pin loosening and subsequent loss of fixation.

Future research and emerging surgical techniques

Significant advancements in imaging and diagnostic modalities are underway, including the integration of artificial intelligence and machine learning to enhance diagnostic precision. This progress holds promise for improved preoperative planning and outcomes [63]. Furthermore, personalized treatment algorithms are emerging, enabling clinicians to tailor treatment strategies to individual patients based on factors influencing optimal outcomes.

Three-dimensional (3D) printing technology has revolutionized PHF management by enabling the creation of patient-specific implants tailored to unique anatomical features, improving implant fit, and potentially reducing complications [64]. Augmented reality (AR) and virtual reality (VR) are increasingly used in preoperative planning, allowing surgeons immersive visualization of fracture patterns and practice in procedures, which ultimately enhances surgical precision. Nanotechnology, through nanostructured surfaces on implant coatings, promotes improved biocompatibility and osseointegration, reducing the risk of complications [65].

## Conclusions

Proximal humerus fractures are a prevalent orthopedic injury, especially among older adults. Understanding the underlying anatomy and using various classification systems are critical for accurate diagnosis and effective treatment planning. Treatment choice-whether conservative or surgical-depends on factors such as fracture pattern, patient age, and functional demands.

Surgical intervention offers potential for improved functional outcomes but is associated with possible complications. Non-surgical management, though less invasive, may have limitations in achieving optimal results, particularly in complex fractures. A patient-centered approach that considers individual factors like comorbidities and functional status is essential, especially in managing fractures in frail elderly patients. A multidisciplinary team involving orthopedic surgeons, physiatrists, and physical therapists ensures comprehensive patient care.

Future research should further explore these technologies’ potential in proximal humerus fracture management, including long-term outcomes of specific surgical approaches and how emerging techniques can enhance patient recovery. Continued advancements in surgical methods and rehabilitation protocols, grounded in these innovations, are likely to further improve management and outcomes in proximal humerus fractures.
